# Immuno-reactive cancer organoid model to assess effects of the microbiome on cancer immunotherapy

**DOI:** 10.1038/s41598-022-13930-7

**Published:** 2022-06-15

**Authors:** Ethan Shelkey, David Oommen, Elizabeth R. Stirling, David R. Soto-Pantoja, Katherine L. Cook, Yong Lu, Konstantinos I. Votanopoulos, Shay Soker

**Affiliations:** 1 Wake Forest Institute for Regenerative Medicine, Winston-Salem, NC 27101 USA; 2 Wake Forest School of Medicine, Winston-Salem, NC 27157 USA; 3 Wake Forest Baptist Medical Center, Winston-Salem, NC 27101 USA; 4 Current Address: Rutgers New Jersey Medical School, Newark, NJ 07103 USA; 5 Current Address: Houston Methodist Research Institute, Houston, TX 77030 USA

**Keywords:** Tissue engineering, Breast cancer, Immunotherapy, Applied microbiology

## Abstract

Immune checkpoint blockade (ICB) therapy has demonstrated good efficacy in many cancer types. In cancers such as non-resectable advanced or metastatic triple-negative breast cancer (TNBC), it has recently been approved as a promising treatment. However, clinical data shows overall response rates (ORRs) from ~ 3–40% in breast cancer patients, depending on subtype, previous treatments, and mutation status. Composition of the host-microbiome has a significant role in cancer development and therapeutic responsiveness. Some bacterial families are conducive to oncogenesis and progression, while others aid innate and therapeutically induced anti-tumor immunity. Modeling microbiome effects on anti-tumor immunity in *ex vivo* systems is challenging, forcing the use of *in vivo* models, making it difficult to dissect direct effects on immune cells from combined effects on tumor and immune cells. We developed a novel immune-enhanced tumor organoid (iTO) system to study factors affecting ICB response. Using the 4T1 TNBC murine cell line and matched splenocytes, we demonstrated ICB-induced response. Further administration of bacterial-derived metabolites from species found in the immunomodulatory host-microbiome significantly increased ICB-induced apoptosis of tumor cells and altered immune cell receptor expression. These outcomes represent a method to isolate individual factors that alter ICB response and streamline the study of microbiome effects on ICB efficacy.

## Introduction

Immune checkpoint blockade therapy (ICB) represents a promising development in treatments for cancer patients. Administration of ICB can assist adaptive anti-tumor reactivity through several mechanisms, including the preservation of dysfunctional T-cells and blocking the immune inactivation pathways utilized by cancer cells to avoid immune-induced cytotoxicity^1^. When used in combination, ICB can elicit particularly potent and distinct actions compared to monotherapy^2^. These therapies even show promise to treat more aggressive disease states in tumors that were previously thought to contain relatively low immune activity^3^. More aggressive breast cancers, such as Basal-like, triple-negative breast cancer (TNBCs), have limited treatment options and often metastasize with fatal consequences. One early study with the anti-PD-L1 antibody avelumab showed an overall response rate (ORR) of only 4.8% in breast cancer patients; however, patients prescreened for PD-L1 expression in a different study had an ORR that reached 21.4%^4,5^. Encouragingly, a different single-arm study demonstrated an ORR up to 43% in metastatic TNBC patients treated with anti-PD-1 and anti-CTLA-4 combination therapy^3,6^. This is a relatively high ORR compared to other studies, but still fewer than half of the patients demonstrated a positive outcome. This incomplete response in selected cohorts shows how specific type of ICB, previous treatments, and PD-1/PD-L1 expression status are not the only factors that have been shown to alter tumor sensitivity to immunotherapy. Accounting for all these factors still can still not fully predict how a patient will respond to ICB. It is therefore vital that the various other factors effecting ICB therapy efficacy be characterized to provide patients with the highest chances of successful treatment outcome.

One extrinsic factor gaining attention is the role of the host-microbiome on ICB response^7–9^. Understanding of the microbiome has progressed greatly over the past few decades. Previously, most of the focus was on only a select few species and their effects on the immediate gut environment where most bacteria are found. Only recently has there been an improved understanding of the many different microorganisms and their effects on treatment in distal organ systems^10^. New studies even show the role of diet on the breast microbiome, ICB efficacy, and local tumorigenesis^11–13^. The presence of different bacterial families, which can be caused by diet, environment, age, pathogen exposure, and more, can prime patients to favorable responses or treatment resistance^14–16^. The effect of the different taxa on ICB response is primarily mediated through the release of various bacterial metabolites; however, thus far, there has been limited research into how these distinct metabolites affect ICB response^8^. The interaction between these metabolites and the host immune system is key to understanding how the microbiome can alter ICB efficacy. This also means that specific positive indicator metabolites could be used as a predictive measure of ICB response^17^. Therefore, it is highly advantageous to create a flexible, high-throughput model that can examine the roles of multiple specific metabolites in ICB response.

Tumors do not consist of merely cancerous cells but comprise a tightly surrounded tumor microenvironment (TME), made up of both the non-cellular extracellular matrix (ECM) and non-cancerous cells. Traditional in vitro 2-dimensional (2D) cell cultures in plastic dishes fall short of replicating the complex in vivo 3D tissue organization and cannot fully replicate the cell–cell and cell-ECM interactions and, importantly, drug diffusion and treatment efficacy. These deficiencies may further place selective pressure on cells, which can manifest as substantial changes in phenotype. In recent years, we have made considerable progress towards building mini-organ (organoid) models that recapitulate cell–cell, cell-extracellular matrix (ECM), and mechanical interactions inside them^18–24^. These organoids represent a system that maintains the scalability and experimental control of an ex vivo model^25^. Importantly, tumor cells inside the tumor organoids have a different phenotype and show different responses to chemotherapy compared with 2D cultures and tumor cells embedded in 3D gels. Patient-derived tumor organoids (PTOs) offer a unique model for studying patient-specific cancers in vitro. These models allow easy manipulation of numerous specific TME factors independently. This system is therefore ideal for screening several bacterial metabolites in a highly controlled environment while maintaining a physiologically relevant immunoreactive model.

In the current study, we created a novel immunoreactive organoid system that combined murine 4T1 TNBC cells and matched activated splenocytes encapsulated in extracellular matrix hydrogels. Analysis first demonstrated the effects of the ICB treatment in our system, validating the immunoreactive nature of our model, with further results showing a cooperative effect between ICB and bacterial metabolites sourced from species that are thought to be positive indicators of ICB response. These results lay the basis for a screening model that could be used to rapidly identify positive and negative effector species and then follow up on how they mechanistically influence ICB therapy.

## Results

### Immune reactive tumor organoids for examining the effects of bacterial metabolites on ICB response

**Figure 1:**
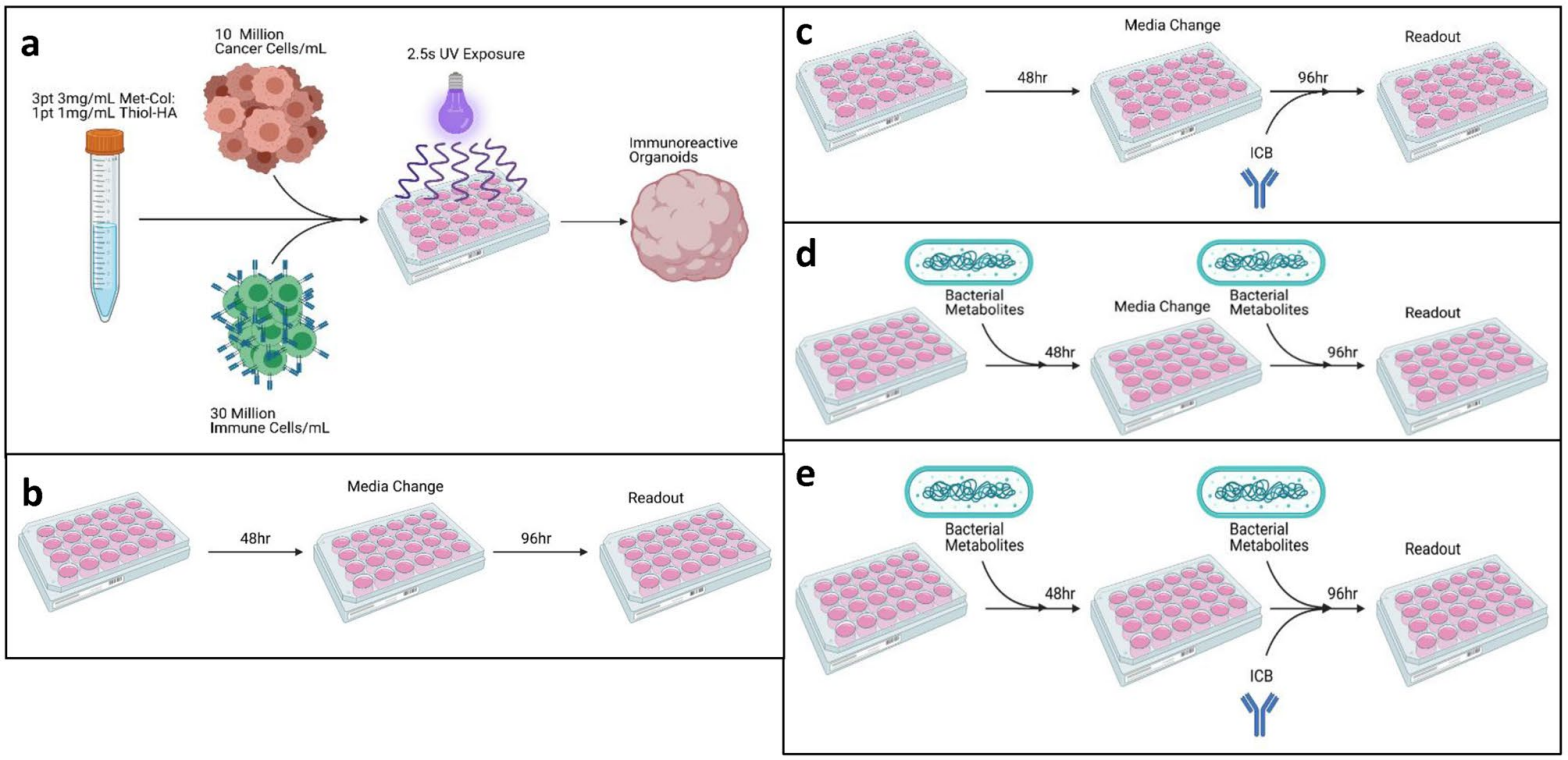
Organoid formulation and general experimental layout. (**a**) Schematic for the construction of the organoids. This equates to 300,000 immune cells and 100,000 cancer cells in each organoid (**b**) Control group that is not treated with bacterial metabolites or ICB for the 5-day course of the experimental trial. (**c**–**e**) Organoids are treated with ICB alone (**c**), bacterial metabolites alone (**d**), and a combination of ICB and metabolites (**e**).

There are components in a tumor model system required to enable adaptive anti-tumor immune response. First, a viable immune cell population, such as cytotoxic T-lymphocytes (CTLs), that can react with the specific antigens presented by the cancer cells must be present. Second, activation of CTLs requires intracellular contacts, which allows the T-cell receptor to directly engage with the antigen presented by the major histocompatibility complex (MHC) on the targeted cancer cell. Third, additional co-receptors such as CD8 must also be directly stimulated for viable targeted immune activity. To recreate this model system ex vivo, our lab utilized a specialized extracellular (ECM) mimicking hydrogel mixture to encapsulate immune and cancer cells (Fig. 1a)^26^. This mixture enables rapid gelation that is especially beneficial for reducing exposure of the relatively sensitive immune cells to short stress conditions while the hydrogel sets. 4T1 tumors present the endogenous retroviral protein gp70 containing the CD8 + T-cell AH-1 epitope^27^. CD8 + T cells derived from 4T1 tumor-bearing mice allow the creation of immune-reactive tumor organoids utilizing this antigen.

**Figure 2:**
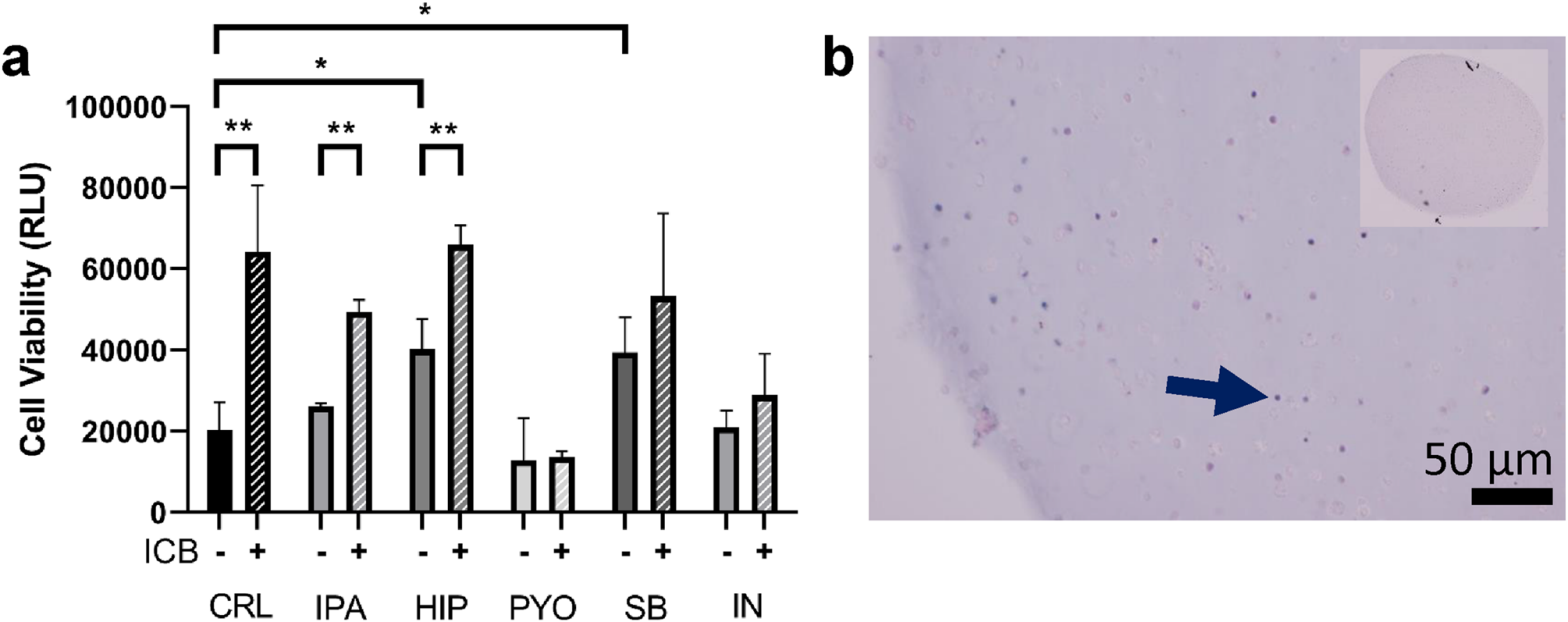
Effect of ICB and bacterial metabolites cell viability in immune cell organoids. (**a**) ATP production was measured via CellTiter-Glo 3D™ for untreated CRL organoids, metabolite treated organoids, ICB treated organoids, and organoids that received a combination of both treatments (n = 4). (**b**) H&E stained splenocytes organoid (arrow indicates splenocyte. 20 × magnification, inset 4 × magnification).

Preliminary experiments focused on the direct effects of bacterial metabolites on these immune cells in the iTOs. In contrast, later experiments assessed the effects on educated lymphocytes combined with 4T1 murine breast carcinoma cells. A baseline of cellular phenotype and behavior was established using organoids cultured in base culture media (Fig. 1b). Then organoids were cultured in the presence of ICBs, (Fig. 1c), or exposed to a single bacterial metabolites listed in Fig. 2, for the full 5-day experiment (Fig. 1d). Metabolites examined include 3-indolepropioninc acid (IPA), hippuric acid (HIP), pyocyanin (PYO), butyrate (SB), and inosine (IN). Each of these metabolites are sourced from species hypothesized to be beneficial to ICB response and administered at concentrations that approximate systemic circulation measurements^28–32^. Finally, the two treatment courses were combined to expose the organoids to ICB and a metabolite (Fig. 1e).

### The combined effect of ICB and bacterial metabolites on immune cell only organoids

To determine the independent effects of ICB and bacterial metabolites on immune cells in the organoids, immune cell-only organoids were constructed with 3 × 10^7^ splenocyte cells/mL in 10 µL organoids. The organoids received ICB (anti-CTLA-4 and anti-PD-1 combination) alone and in combination with different bacterial metabolites, as listed in Fig. 2a. Immune cell viability, as determined using CellTiter-Glo 3D™, a cellular ATP assay, after 5 days in culture, demonstrated a response to both ICB and metabolite treatment (Fig. 2a). ICB treatment alone significantly increased cell viability (*p* = 0.0026). Treatment with HIP and SB alone increased immune cell viability compared to an untreated sample (*p* = 0.032 and 0.0128). ICB combinations with IPA and HIP showed no significant changes compared with ICB alone but were significantly greater than their respective metabolite-only treatment (*p* = 0.0007 and *p* = 0.0162). However, PYO and IN prevented the ICB-induced increase in immune cell viability and had no increase in ICB untreated viability either. In parallel, organoids were fixed, sectioned, and stained with hematoxylin and eosin (H&E), revealing immune cells interspersed randomly throughout the hydrogel (Fig. 2b). Taken together, bacterial metabolites such as HIP and SB may increase immune cell proliferation while PYO and IN prevented the ICB-induced increase in immune cell viability, suggesting not only the direct effect of some bacterial metabolites on immune cell viability but also their ability to impact the effects of ICB treatment.

### Characterization of immune reactive tumor organoids (iTOs)

**Figure 3:**
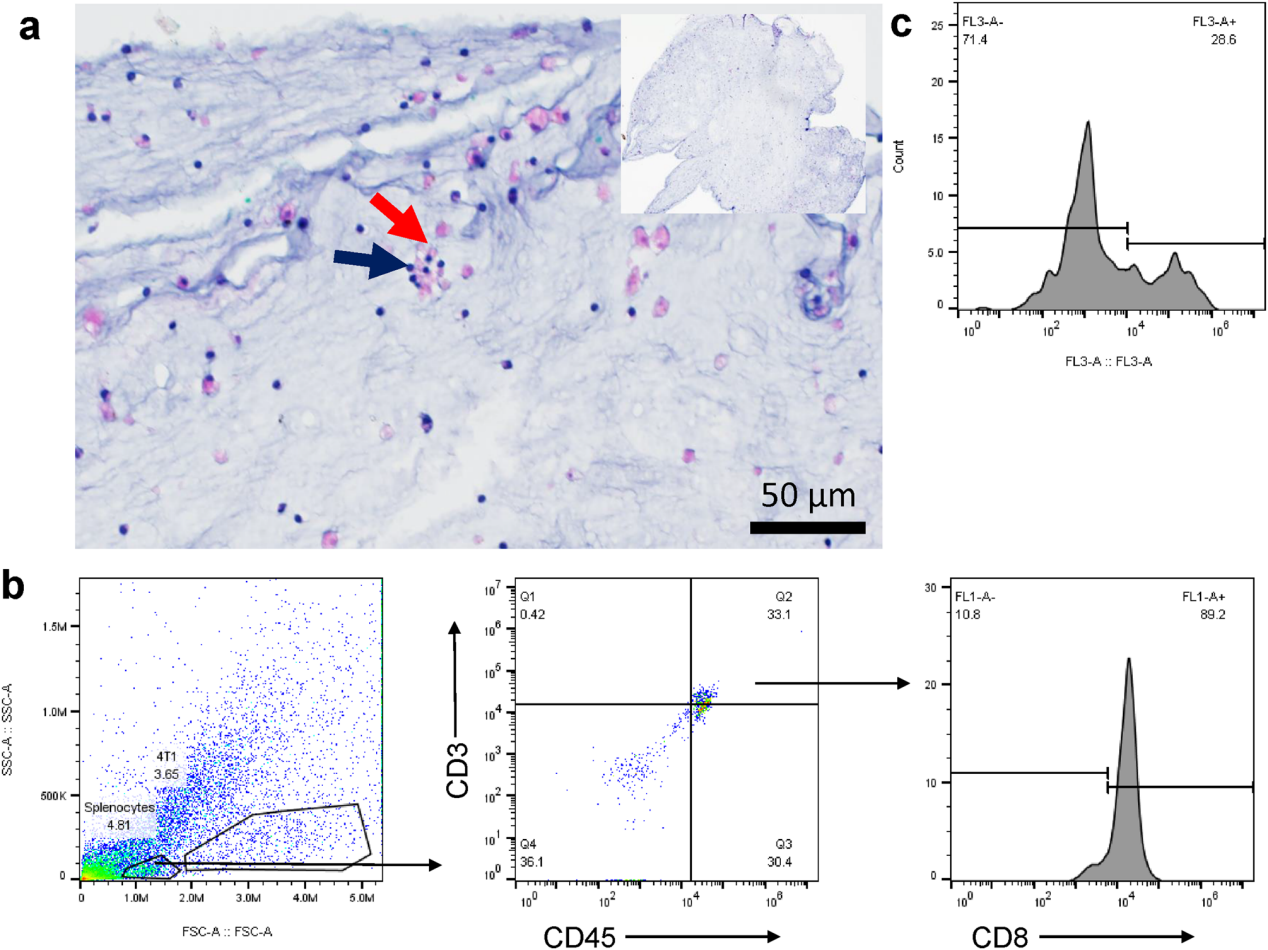
Characterization of iTOs. (**a**) H&E stained iTO shows immune cell co-localizing to cancer cells (purple arrow indicates splenocytes and red indicates cancer cells. 20 × magnification; inset 4 × magnification). (**b**) Flow cytometry analysis of broad FSC-A vs. SSC-A, CD45^+^CD3^+^_,_ and CD45^+^CD3^+^CD8^+^cells isolated from the iTOs., as indicated (**c**) Viability of CD45^+^CD3^+^CD8^+^ cell fraction where the Y-axis is cell count and the X-axis is fluorescent intensity (n = 3 organoids).

We next characterized organoids in which the immune cells were co-encapsulated with 1 × 10^7^ 4T1 murine TNBC cells/mL. The end result was 10 µL organoids containing 3 × 10^5^ activated splenocytes and 1 × 10^5^ 4T1 cells, a 3:1 effector to target cell ratio. Histological examination of iTOs revealed a clustering of the immune cells around the cancer cells (Fig. 3a). This is, in contrast, to randomly dispersed immune cells in immune cell only organoids (Fig. 2b).

Flow cytometry was performed to further characterize the immune population in the organoids. The chemical cross-linking of the hydrogel in the organoids resulted in good physical properties that supported high immune cell viability but increased the difficulty of single-cell isolation. This was overcome using a combination of hyaluronidase and collagenase I, III, and IV. After straining the digested organoids preparation through a 40 µM mesh, cells were stained with CD45, CD3, and CD8 antibodies to quantify the immune cell fraction in the iTOs (Fig. 3b). All gating for cell populations was performed against appropriate isotype controls (Supplementary Fig. S1). Results demonstrate that 33.1% of the isolated cells were CD45^+^CD3^+^ T-cells, of which 89.2% were CD8^+^ cytotoxic T-cells. Negative propidium iodide staining showed 71.4% viable CD8^+^ cells under our culture conditions (Fig. 3c). These results suggest that the iTOs maintained a viable CTL population that could localize and contact the 4T1 tumor cells.

### ICB increases immune cell viability and cytotoxicity in iTOs

**Figure 4:**
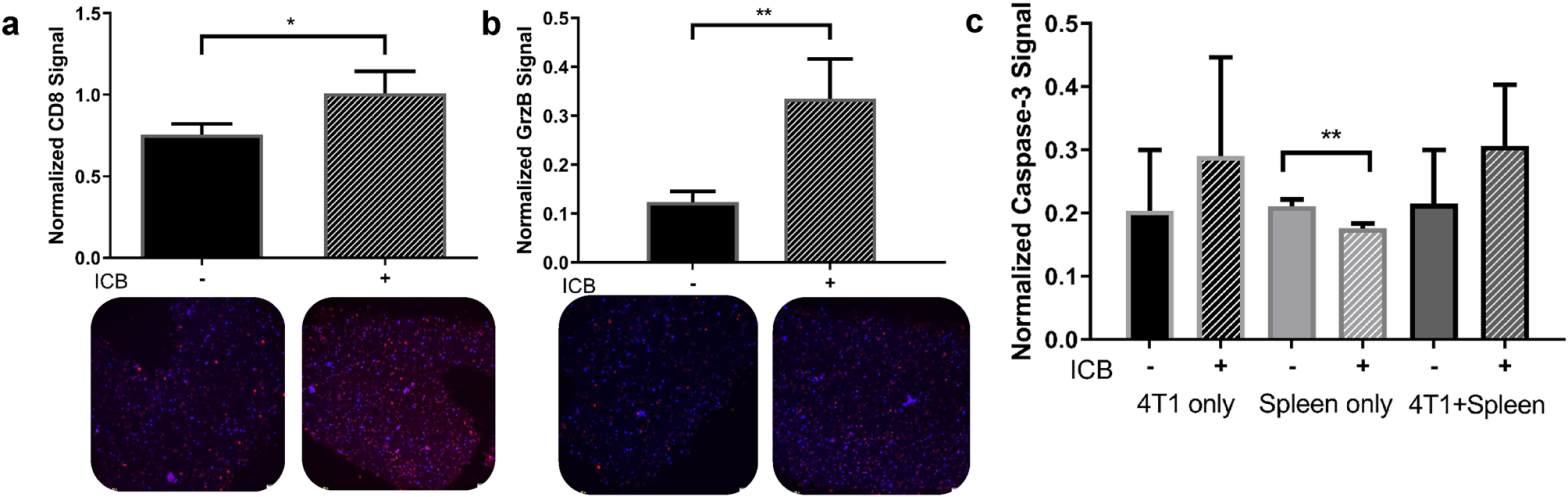
The effects of ICB on immune cell cytotoxicity in iTOs. ITOs were treated with anti-CTLA-4 and anti-PD-1, ICB combination, as indicated, and analyzed for CD8, granzyme B, and cleaved caspase 3 expression with immunostaining. (**a**, **b**) DAPI normalized CD8-positive (**a**) and granzyme B-expressing (**b**) cell numbers are significantly increased by ICB administration in iTOs. (p = 0.0158 and 0.0024 for CD8 and granzyme B, respectively, n = 4). Representative IF images from each group are shown below. (**c**) Cleaved caspase-3 IF signal in 4T1 cell only organoids, splenocytes only organoids and iTOs, treated with ICB and untreated, as indicated (significance in splenocytes only organoids p = 0.0021) (n = 4 ROI).

We next tested the effects of anti-CTLA-4 and anti-PD-1 in combination, which has proven effective in TNBC clinical trials, on immune cell viability and cytotoxicity in our combined cell iTOs^6^. Exposure of iTOs to ICB caused a significant increase in the numbers of CD8-positive cells and granzyme B production when normalized to cell number (DAPI) and compared to untreated samples (*p* = 0.0158 and *p* = 0.0024) (Fig. 4a,b). Cleaved caspase 3 signal, indicative of cellular apoptosis, was increased in iTOs containing tumor and immune cells, albeit not significantly (Fig. 4c). In contrast, there was a slight but significant decrease in cleaved caspase 3 in the immune cell only organoids (*p* = 0.0021) (Fig. 4c). Taken together, these results demonstrate that administration of ICB to iTOs increases immune cell numbers, anti-tumor cytotoxicity, and tumor cell apoptosis.

### The effects of bacterial metabolites on the tumor immune microenvironment

The host microbiome significantly modulates the immune environment both proximally to the gut where most microbes are found and in more distal areas with their own smaller microbiome population^33–35^. Breast tissue is one example where the local microbiome and metabolites in the blood from other sites elicit modulatory effects. Evaluation of the role of pharmacologically active bacterial metabolites on the tumor immune environment will allow a better understanding of the role of each distinct family of bacteria in modulating tumor response to ICB therapies.

**Figure 5:**
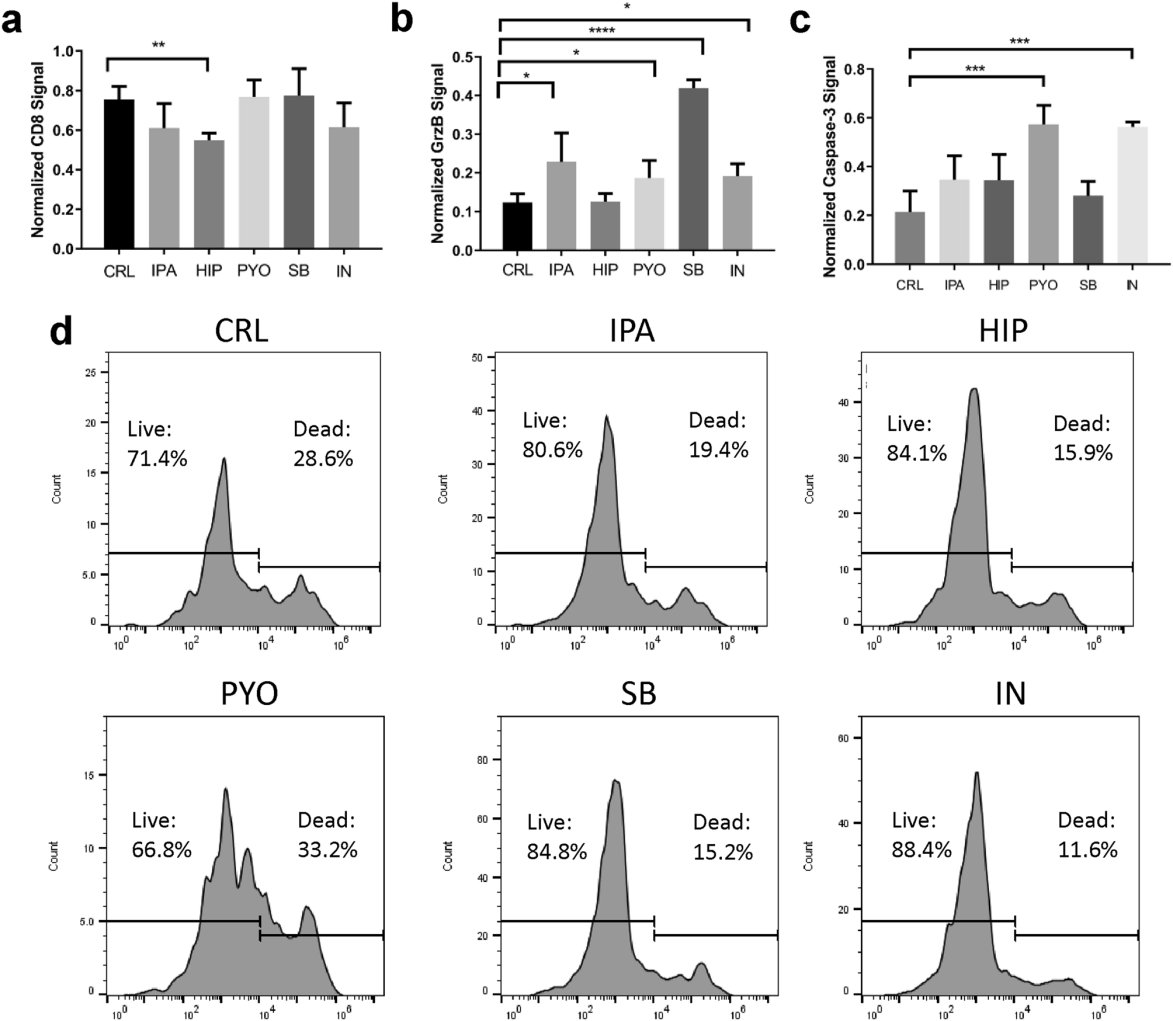
The effects of bacterial metabolites on immune responses in iTOs. (**a**–**c**) CD8 (**a**), Granzyme B (**b**), and cleaved caspase-3 signals (**c**) normalized to DAPI signal in iTOs (n = 4 ROI). (**d**) The effects of bacterial metabolites on CD8^+^ immune cell population viability (data representative of 3 organoids combined).

To replicate the effects of metabolites in a distal site, each metabolite was administered into the culture media of the iTOs at physiological circulating concentrations. The metabolites were present ubiquitously for the course of the experiment (in the initial culture medium and added 48 h later), and organoids were evaluated for CD8, granzyme B, and cleaved caspase-3 signals. Additionally, the CTL population viability was measured using flow cytometry. Immunofluorescence (IF) quantification showed that several of the metabolites elicit heightened immune action (Fig. 5a–c). CD8 signal was unchanged or significantly decreased by HIP (*p* = 0.0014, n = 4) (Fig. 5a). On the other hand, granzyme B signal was elevated upon exposure to IPA, PYO, SB, and IN (*p* = 0.0379, 0.0470, *p* < 0.0001, and 0.0133, respectively) (Fig. 5b), and increased cleaved caspase-3 signal was seen upon exposure to PYO and IN (*p* = 0.0008, *p* = 0.0002) (Fig. 5c).

While some of the metabolites increased overall apoptosis in the iTOs, as measured by caspase-3 signal, it was essential to determine that this result was not due to loss of CTL viability, as that would be counterproductive to ICB efficacy. CD8^+^ cell survival, as measured via negative propidium iodide staining, was 71.4% in untreated iTOs, and was increased in IPA, HIP, SB, and IN treated organoids to 80.6%, 84.1%, 84.8%, and 88.4%, respectively (Fig. 5d). On the other hand, PYO treated organoids had a reduced 66.8% CD8^+^ cell survival (Fig. 5d). These increases in CTL viability over the short 5-day time frame indicate that the bacterial metabolites do not have adverse effects on CTL in the iTOs, similar to immune-only organoids.

### Bacterial metabolites act cooperatively with ICB to elicit stronger anti-tumor responses

**Figure 6:**
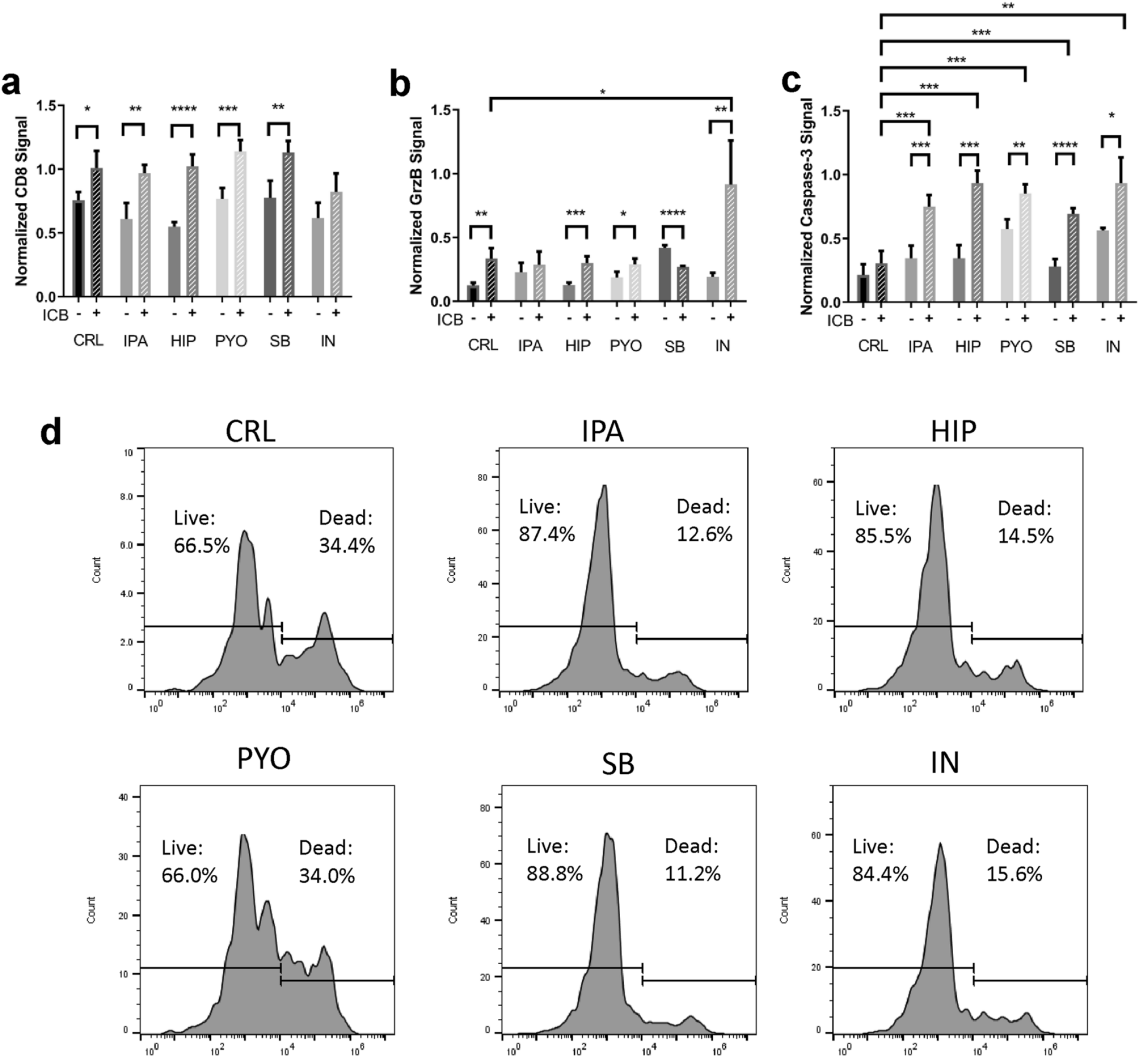
The effects of bacterial metabolites and ICB combinations on immune cell cytotoxicity in iTOs (**a**–**c**) CD8 (**a**), Granzyme B (**b**), and cleaved caspase-3 signals (**c**) normalized to DAPI signal in iTOs (n = 4 ROI). (**d**) Viability of the CD8^+^ immune cell population in the different metabolite treated samples, all samples treated with ICB (data representative of 3 organoids combined).

In each patient, the immune system constantly interacts with the byproducts and metabolites from the microbiome. Exposure of the iTOs to ICB and metabolites separately (Figs. 4, 5, respectively) demonstrated increased viability and cytotoxicity of CTLs. Synergistic results of ICB and metabolites in the iTOs could indicate a potential for therapeutic convergence between the ICB and the microbiome. To test for such effects, iTOs were made as described before, and after the initial 48-h exposure to the metabolites, the media was replaced with media containing both ICB and metabolites. Similar histological (IF) and flow cytometry assessments were made at the end of the experiment (Fig. 6a). CD8^+^ expression was heightened over untreated, and metabolite only control groups upon exposure to ICB alone and ICB in combination with IPA, HIP, PYO, and SB (*p* = 0.0023, *p* < 0.0001, *p* = 0.0010 and *p* = 0.0048, respectively). Interestingly, exposure to ICB and HIP combination rescued the decrease in CD8^+^ signal seen with exposure to HIP alone (Fig. 5a). Granzyme B signal was significantly higher in iTOs exposed to ICB in combination with HIP, PYO, and IN when compared with metabolite-only exposure (*p* = 0.0008, *p* = 0.0172, *p* = 0.0058, respectively), but not when compared with ICB alone treatment. On the other hand, exposure to ICB and IN combination resulted in a significantly higher granzyme B signal compared with ICB alone treatment (*p* = 0.0169) (Fig. 6b). Cleaved caspase 3 signal was the most affected by co-administration, with significantly higher signal in all combinations of the bacterial metabolites and ICB compared to either metabolite or ICB treatments alone (Fig. 6c) (metabolites: *p* = 0.0009, *p* = 0.0002, *p* = 0.0020, *p* < 0.0001, *p* = 0.0107 for IPA, HIP, PYO, SB and IN, respectively; ICB: *p* = 0.0005, *p* = 0.0001, *p* = 0.0001, *p* = 0.0004, *p* = 0.0014). Taken together, these results clearly demonstrate that the combination of ICB with metabolites from most bacterial species tested here has a marked additive effect on immune cell cytotoxicity against tumor cells. We further measured CD8^+^ T-cell viability to ensure that the apoptotic signal was not derived from CTL cell death. A similar pattern emerged as in the metabolite-only treated iTOs (Fig. 5d). Except for PYO, the other metabolites IPA, HIP, SB, and IN increase CD8^+^ cell survival, as measured via negative propidium iodide staining by ~ 25–30% (Fig. 6d). This result further demonstrates the role of the metabolites in preserving the immune cell population in the iTOs and their potential to aid in ICB response.

### Bacterial metabolites and ICB combinations induce cancer inflammation and immunity mRNA expression

**Figure 7:**
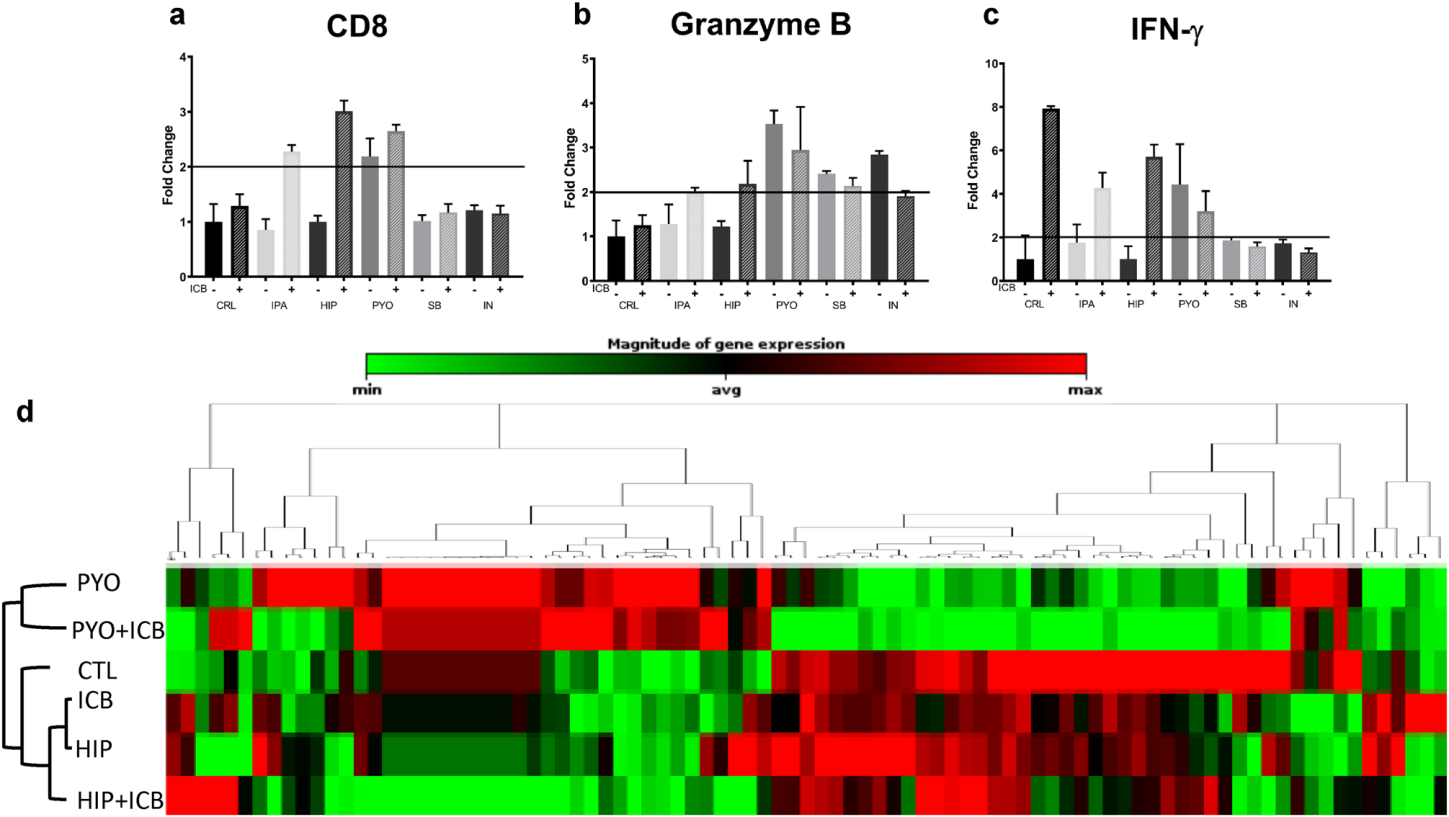
The effects of bacterial metabolites and ICB combinations on cancer inflammation and immunity mRNA expression (**a**–**c**) mRNA analyzed for CD8 (a), Granzyme B (b), and IFN-γ (c). (**d**) Heatmap of a Mouse Cancer Inflammation and Immunity RT^2^ qPCR Profiler PCR Array™ performed on CRL, HIP, and PYO treated organoids with and without ICB, as indicated. Color gradient is relative across all samples and genes shown (n = 3 organoids).

Bacterial metabolites have significantly affected immune cell function and phenotype in the iTOs. Different metabolites impact protein expression differently and further alterations upon the combination with ICB treatment. To quantify these effects, mRNA was extracted from iTOs at the end of the treatment and analyzed for changes in the expression of cancer inflammation and immunity genes. Co-treatment of iTOs with IPA, HIP, and PYO with ICB showed an increase of greater than a twofold expression of CD8 at the final time point (Fig. 7a). This continues to support the hypothesis that the combination of treatment and metabolite can produce a more durable effect than either the metabolite or ICB independently. Granzyme B mRNA expression was increased in PYO, SB, and IN treated iTOs without ICB (Fig. 7b). ICB did not further increase Granzyme B mRNA expression. Examination of IFN-γ mRNA expression to further assess immune function demonstrated an increase in ICB-only treated iTOs (Fig. 7c). The combinations of ICB with IPA and ICB with HIP increased IFN-γ mRNA expression compared with untreated iTOs and metabolite only treated iTOs, however, to a lesser extent than ICB only treatment (Fig. 7c). To get a better understanding of the scale of expression alteration, mRNA from untreated iTOs, PYO only, and HIP only treated iTOs and mRNA from iTOs treated with ICB only and ICB in combination with PYO and HIP were analyzed using a profiler array focusing on immune activation better to illustrate the genotypic effects (Fig. 7d). Results showed mRNA expression varies depending on the metabolite administered. While the control and HIP treated iTOs were relatively similar in terms of mRNA expression alterations regardless of combination with ICB, the PYO and PYO + ICB treated iTOs showed a vastly different expression profile. Closer examination of PYO treatment-specific mRNA expression showed a significant increase in FAS-ligand mRNA and increased Granzyme A and B production, suggesting a potential mechanism for the increased cytotoxicity in the iTOs. The array also shows HIP dependent downregulation of immunosuppressive cell recruiting chemokine receptors CXCR1 and CXCR2, and anti-inflammatory interleukins IL-10, IL-13, and IL-17a. There were a number of other potentially relevant significant fold changes in mRNA expression recorded (Supplementary Fig. S2). Taken together, these observations may outline the mechanisms behind the effects of bacterial metabolites on ICB treatment results in the iTOs.

## Discussion

It is well accepted that the host microbiome has a significant role in carcinogenesis and response to cancer therapies, especially immunotherapies^9,12,14,17,36,37^. However, it is difficult to isolate the effects of singular metabolites in vivo due to the presence of a large variety of distinct species of bacteria from different families. These species may produce factors that have competing effects on the host immune system and may obfuscate direct relationships between the metabolites and the therapeutic outcomes. The goal of the current study was to assess the potential of immune-enhanced tumor organoids (iTOs) to evaluate the direct effects of different bacterial metabolites on immune cells separately and the efficacy of immunotherapy in a combined matched immune tumor cell organoid environment. To achieve this goal, we created a novel iTO system specifically designed to produce a viable immune interaction between murine 4T1 TNBC and matched activated splenocytes. The specific mixture of chemically altered hydrogels was selected to minimize cellular exposure to unfavorable conditions, media was formulated to allow proper unhindered immune cell function, and seeding density was managed to prevent overly necrotic cores that could act as a physical barrier to immune action. This system was used to model the phenotypic, cytotoxic, and genotypic alterations caused by host-microbiome metabolites in cancer patients, and especially the effects on ICB outcomes, a therapy that has the potential to greatly aid patients but shows limited overall effectiveness in clinical settings. The methods employed by this study to create an immunoreactive environment are only replicated in one other study from our lab^38^. A key difference between this model and other organoids that incorporate immune elements is that other groups often utilize peripheral blood mononuclear cells or the tumor-infiltrating T-cell population in the formulation^38–40^. Our model uses antigen-specific primed T-cells derived from splenocytes, allowing for a high immune cell yield that is also relatively immunoreactive. This study specifically examines the behavior of the CTL population in these splenocytes and their response to stimuli in our iTO system because the CTL population is a predominant target affected by ICB and is responsible for much of the elicited cytotoxicity^1^.

Using the iTOs model system, we were able to demonstrate the effects of key microbiome metabolites on immune function and therapy in the local TME. Microbial metabolites sourced from species theorized to aid ICB response were shown to upregulate critical elements in the immune response pathway. Furthermore, when these elements were combined with ICB, there was a cooperative effect in anti-tumor cell activity and preservation of immune cell viability. Our results show that iTOs demonstrate an interplay between microbiome-derived metabolites and host immune cells and the resulting impact on immunotherapy. Generally, administration of systemic concentrations of selected bacterial metabolites, which can be seen in cancers distal from the gut, increased the numbers and viability of CD8^+^ T cells and increased production of granzyme B.

Similarly, ICB treatment of iTOs increased immune response markers. Specifically, we found that when administered together with ICB, specific metabolites could increase CD8 expression, cooperatively work to decrease cancer cell viability, and increase CTL survival. mRNA analysis revealed some metabolites and ICB induced upregulation in key immune factors, such as CD8 and IFN-γ, which mimics results from in vivo analysis^8^. Results from the RT^2^ profile array demonstrate the dramatic extent to which metabolites can alter genetic expression. Together, these results corroborate in vivo experiments and validate the iTO as a physiologic facsimile representing the in vivo state of the TME^36^. Further understanding the relationship between specific metabolites and overall response to ICB could be used to screen for positive potential outcomes. Procedures such as fecal transfer or diet modification could also effectively induce a more conducive microbiome for therapy^41,42^.

Each of the five different metabolites examined differ significantly in molecular structure and function. SB is a short-chain fatty acid (SCFA), and in agreement with the data provided here, previous studies have shown that SCFA molecules can induce apoptosis in tumor cells as assessed by cleaved caspase-3^43^. The mechanism proposed for this activity is signaling through the apoptosis regulating G protein-coupled receptors GRP41 and GRP43. Knockdown of these GPRs lead to reduced caspase cleavage. However, the role of GPR41 and GPR43 in disease and treatment is inconsistent in the literature, and signaling via these receptors is described as both beneficial and detrimental to cancer therapy. There are also questions about the differences between non-human and human functionality. More data is required to determine the relationship between these receptors, the bacterial metabolites, and therapeutic response. Further use of this model with patient-derived cells instead of murine-sourced samples will help demonstrate the full effects of these factors. Other ICB studies examining the effects of SCFA studies show the direct inverse result of the described findings, pointing to bacterial sourced SCFA such as SB or propionate as mitigating factors working against anti-CTLA-4 ICB^44^. The mechanism proposed for this is an increase in Tregs fueled by SCFA metabolism, decreasing effector immune cell activity. However, our profiler array shows no significant increase in the Treg-related FOXP3 after metabolite exposure. While the overall cytotoxicity results differ, mRNA from our organoid experiment and Coutzac et al. show mitigation of IFN-γ expression in the presence of SB. While other trials found the nucleoside inosine to upregulate CTL IFN-γ, RNA results from this trial show little effect^8^. Meanwhile, our results agree with other findings that the combination of ICB and HIP can increase IFN-γ for anti-tumor immunity^45^. Our inflammation array also showed HIP dependent downregulation of several factors that are associated with an immunosuppressive environment. HIP decreased expression of CXCR1 and CXCR2 mRNA, which are chemokine receptors that recruit myeloid derived suppressor cells (MDSC) and are correlated with poor prognosis^46^. HIP also decreased MDSC recruiting IL-17 and immunosuppressive IL-10 and IL-13^47,48^. Finally, the relationship between ROS and anti-tumor immunity is known to be complicated with conflicting outcomes from different ROS-producing and scavenging agents; however, there is little genetic data in the literature illustrating the role of the producer PYO or the scavenger IPA on ICB^49^. Data from the profiler array suggests that ROS introduction by PYO upregulates FAS-L on the CTLs, leading to increased targeted anti-tumor apoptosis.

The current study yielded promising results, including developing immune-reactive tumor organoids (iTOs) model system and the additive effect of selected metabolites on ICB-induced tumor cell apoptosis. The iTO model is still in development and is not ready for immediate translation to prediction of clinical outcomes in cancer patients. Our iTOs specifically focus on the role of CTLs in ICB response but are still lacking characterization of other cell types contributing to the immune response, such as macrophages, that could play an important role in the clearance of both cellular debris and possibly the metabolites themselves. An ideal model would also benefit from a multilevel analysis when determining the actual therapeutic value for each patient. Targeted genomic analysis and broad array analysis will determine the full extent to which extrinsic factors, which have not been accounted for until recently, can impact the therapeutic efficacy. Yet, the results suggest that further development of this model will eventually be an important clinical tool in designing and analyzing future trials for anti-cancer treatments. We are currently applying the results in murine breast cancer iTOs to human breast cancer patient candidates for immunotherapy, with a goal to integrate the iTOs platform in the treatment decision process.

## Methods

### Murine 4T1 TNBC cells and murine splenocytes

All animal procedures were approved and performed according to the Wake Forest School of Medicine All procedures were approved and performed in strict accordance with the NIH Guide for the Care and Use of Laboratory Animals, and the policies of the Wake Forest School of Medicine Institutional Animal Care and Use Committee (IACUC). 4T1 murine TNBC cells were obtained from ATCC. This line was chosen because they express retroviral protein gp70^50^, allowing for standardization of cellular in vitro response of harvested splenocytes. 5 × 10^5^ 4T1 cells were injected orthotopically into the mammary fat pads of BALB/c mice (Jackson Laboratory, USA). The mice were sacrificed once their tumors reached a size of 200 mm^3^ over 7–14 days. The mouse spleens were isolated and placed in a 50 mL conical tube with 5 mL RPMI-1640 (ATCC, USA) with P/S (Fischer Scientific, USA), L-Glut (GIBCO, USA), NaPyr, 10 mM HEPES (GIBCO, USA), 50 uM BME (Sigma Aldrich, USA), 1% NEAA (Cyagen, USA), 10% Hy Clone FBS (Thermo Fischer Scientific, USA) at 4 °C while being transported. The spleen capsule was removed, and the remaining tissue was sliced into 2–4 fragments. Each spleen was then incubated in 6 mL of the RPMI-1640 media with 2 mg/mL collagenase type IV (Worthington Biochemical Corporation, USA) for 15 min. Ice cold FBS enriched media was then added at a 1:1 ratio, and the tube was placed on ice to halt the digestion process. The pieces of softened spleen were then placed on a 100 µM cell strainer (Thermo Fischer Scientific, USA) in a 50 mL conical and homogenized and washed with 10 mL of complete RPMI-1640 media. Cells were at 450 g for 5 min, and the media was removed from the pellet. Cells were collected by centrifugation and suspended in 5 mL of 1 × RBC (Biolegend, USA) lysis buffer at 4 °C in the dark for 5 min with occasional agitation. Cells were collected by centrifugation and resuspended at 1–2 million cells/mL or 3–5 million cells/ 2 mL in a 24 well plate in the complete media with the addition of 50 IU/mL murine IL-2 (R&D Systems, USA) and 1 µM gp70 peptide (ANASPEC, USA), which is a fragment of the AH1 antigen found on 4T1 TNBC cells for 5 days. This allows for further growth and differentiation of ant-4T1 cytotoxic T-cells. A total of 12 spleens were utilized from 4T1 TNBC tumor bearing mice.

### Organoid formulation

Immune enhanced tumor organoids (iTOs) were created by encapsulating ~ 10 × 10^6^ tumor cells and ~ 30 × 10^6^ immune cells counted with a Nucleocount NC-200 (Chemometec, Denmark) per 1 mL of a 3:1 mixture of 3 mg/mL methacrylated collagen (Advanced Biomatrix, USA) suspended in 20 mM acetic acid (Advanced Biomatrix, USA) and 1 mg/mL thiolated hyaluronic acid (Advanced Biomatrix, USA) suspended in 0.1% w/v irgacure photo-initiator (Advanced Biomatrix, USA) in autoclaved DO water. The hydrogel mixture was neutralized with 95ul of Neutralization Solution (Advanced Biomatrix, USA) per 1 mL of 3 mg/mL collagen utilized. This leads to a final concentration of 2.25 mg/mL collagen and 0.25 mg/mL collagen in the organoid. 10µL of the mixture (equal to 1 × 10^5^ 4T1, 3 × 10^5^ immune or 4 × 10^5^ combined cells) was pipetted into individual wells in a 48 well plate (Falcon, USA) coated with 120 µL PDMS (DOW Chemical, USA) to prevent cell outgrowth outside the organoids. The hydrogel mixture was exposed to 13.2 J/m^3^ UVA radiation for two and a half seconds to initiate the crosslinking (gelation) of the hydrogel to form the individual organoids. Pervious work with this hydrogel system has demonstrated that no phenotypic and viability changes in cells are observed^19,26,38^. Control organoids were also created with either the immune cells or the tumor cells only.

### Organoid culture and viability testing

Organoids are cultured in 350 µl RPMI 1640 with 1% P/S, 1 mM sodium pyruvate, 4 mM L-glutamine, 10 mM HEPES, 50 µM β-mercaptoethanol, 1% NEAA, 10% HyClone FBS, and 50 IU/mL murine IL-2. Control groups will have a single media change at 48 h before being analyzed at the 5-day time point. Some organoids received ICB treatment of anti-CTLA-4 (Bio X Cell, USA) and anti-PD-1 (Bio X Cell, USA), some received bacterial metabolites and some a combination of ICB and single bacterial metabolite. The combination group used to observe the interaction of ICB and metabolites had the metabolites present for the first 48 h alone, allowing for gene expression changes to occur^8^. The ICB was then added at 48 h with the media change that still contained the metabolites being examined. Cell viability was assessed in immune cell only iTOs using the CellTiter-Glo 3D (Promega, USA) assay analyzing cellular ATP.

### Bacterial metabolites

The bacterial metabolite concentrations used were: 1 µM 3-indolepropionic acid (IPA)^17^, derived from the bacterial species *Clostridium sporogenes*, 100 µM hippurate (HIP)^51^, derived from *Clostridiales spp.*, *Faecalbacterium prausnitzii*, and *Eubacterium spp.*, 10 µM pyocyanin (PYO), derived from *Pseudomonas aeruginosa*, 100 µM butyrate (SB)^52–54^, derived from Firmicutes including *Faecalbacterium prausnitzii* and *Clostridium leptum*, and 10 µM inosine (IN)^8^, derived from *Bifidobacterium pseudolongum* All bacterial metabolites were purchase from Sigma Aldrich, USA. The metabolites were first added when the organoids were created and added again in new media at the 48-h media change. Higher concentrations are found in the local gut environment, where most of the bacteria are found. Still, these concentrations do not reflect systemic metabolite levels that would be observed in distal cancer types such as TNBC.

### Immune checkpoint blocking (ICB) treatments

ICBs anti-CTLA-4 and anti-PD-1 were used at a concentration of 5 µg/mL each, which is below the plasma concentration achieved with the standard dose of 3 mg/kg administered to patients^55,56^. This dose was added with new media at 48 h.

### Histological analysis and quantification

At predetermined time point, organoids were washed twice with PBS (Cytiva, USA) and fixed at room temperature for 5 h with 4% PFA (Electron Microscopy Sciences, USA), washed with PBS, and placed in 70% ethanol for no more than four days before paraffin embedding. 5 µm sections were stained with Hematoxylin and Eosin (H&E). For immunofluorescence (IF) staining, first, the slides were deparaffinized and incubated for 1-h with citrate pH 6.0 for antigen retrieval and permeablized with 0.1% v/v Triton-X (Sigma Aldrich, USA) in PBS for 5 min. Following PBS washes and a 30-min incubation with blocking solution (Abcam, USA), primary antibodies were suspended between 1:200 and 1:500 in antibody dilution solution and left to incubate overnight at 4 °C (Abcam, USA). One section per slide was left unstained as control. Slides were then washed 3 × with PBS, and secondary antibodies (Biotium, USA) were added at 1:250 to all sections and incubated for 1 h at room temperature. Slides were washed 3 × with PBS, and then 1:300 DAPI (Invitrogen, USA) diluted in PBS was added for 8 min. Slides were washed 3 × with PBS, and coverslips were mounted with Prolong Gold (Invitrogen, USA) to extend IF signal life. The exposure was kept the same across all slides for each antibody and had to be kept under the exposure where there was no visible signal on the secondary only slide. For quantification, fluorescent images for the DAPI and signal were saved separately. ImageJ was then used to measure the signal intensity of both the DAPI and the stained signal in the same spot within the boundaries of the organoid, with four regions of interest analyzed per sample. The signal intensity from the stain was divided by the intensity from the corresponding DAPI stain to normalize for potential uneven cellular distribution within the organoid. This data was exported to Prism Graphpad for statistical analysis and visualization. Groups were compared against appropriate controls using an Unpaired T-test. Alpha value was set to 0.05.

### Flow cytometry

At the end of the 5-day experiment, 3 organoids were removed from each group and combined into one run to be digested for flow cytometry. Organoids were placed in 500 µL of HBSS containing 25 u/mL Type I collagenase, 100 u/mL Type III Collagenase, 200 u/mL Type IV Collagenase (All Worthington Biochemical Corporation, USA), and 400 u/mL Hyaluronidase (Stemcell Technologies, Canada). The samples were placed on a rotating rack at 37 °C for 10–30 min. The enzyme solution was neutralized with cold RPMI-1640 with 20% FBS. Cells were run through a 40 µm strainer to remove any larger organoid pieces that did not fully digest. Cells were spun at 500 g for 5 min and suspended in cold PBS with 10% FBS. Cells were then spun again, and all groups being stained were suspended in flow staining buffer containing 0.2–4 µg/mL conjugated primary antibody (Biolegend, USA) or isotype control (Biolegend, USA). Cells were incubated at room temperature for 45 min in the dark. Cells were spun again and washed 3 times with cold PBS with 10% FBS. Cells were finally resuspended in 220ul cell staining buffer (Invitrogen, USA). Cells were tested for viability had 1:500 propidium iodide (Invitrogen, USA) in cell staining buffer. 150ul of each sample were read on a BD Acura C6 Plus (BD Biosciences, USA). Data was then exported as FCS files and analyzed in FlowJo V10. Doublet elimination was performed for both forward scatter and side scatter due to a higher level background data from small digested ECM pieces. Gates for stains were determined using appropriate isotype controls. Gates for viability were determined using unstained controls.

### RNA isolation

Two organoids from each group were removed at the end of the experiment for RNA extraction. Cells were isolated using the Invitrogen PureLink RNA Mini Kit (Invitrogen, USA). Organoid structure was disrupted by pulling the organoids in lysis buffer through a 21 g needle 7–10 times until the matrix was dissolved. RNA was quantified and quality controlled with a nano-drop 2000.

### RT-qPCR and RT^2^ qPCR Profiler PCR Array

To get a general overview of gene expression changes, the control group, the hippurate exposed group, and the pyocyanin exposed group were run through the Mouse Cancer Inflammation and Immunity RT^2^ qPCR Profiler PCR Array (Qiagen, Germany). cDNA for this trail was run was created with the RT^2^ Easy First Stand kit (Qiagen, Germany). For all groups listed, both the ICB exposed and control groups were examined. Data was analyzed using Qiagen’s GeneGlobe Data Analysis Center. Further RT-qPCR was performed using the LunaScript RT SuperMix Kit (New England Biosciences, USA) for cDNA, Eurofins Scientific (Luxembourg) to produce the primers, Power SYBR Green Master Mix (Thermo Fischer, USA), and an Applied Biosystems QuantStudio 3 to run the qPCR. Data was analyzed in the ThermoFischer Cloud. qPCR primers: GAPDH- Forward: 5′-AACTTTGGCATTGTGGAAGG-3′ Reverse: 5′-CACATTGGGGGTAGGAACAC-3′; CD8- Forward: 5′-CCGTTGACCCGCTTTCTGT-3′ Reverse: 5′-CGGCGTCCATTTTCTTTGGAA-3′; Granzyme B- Forward 5′-CAGGAGAAGACCCAGCAAGTCA-3′ Reverse 5′-CTCACAGCTCTAGTCCTCTTGG-3′; IFN-γ- 5′-TGAACGTACACACTGCATCTTGG-3′ Reverse 5′-CGACTCCTTTTCCGCTTCCTGAG-3′.

## Supplementary Information

Supplementary Figures.

## Data Availability

The datasets generated and/or analyzed during the current study are available in the NCBI Gene Expression (GEO) repository with the Accession Number GSE196268.

## Abbreviations

CD: Cluster of differentiation
CRL: Control group
CTL: Cytotoxic T lymphocyte
ECM: Extracellular matrix
HIP: Hippurate
IFN-γ: Interferon gamma
IN: Inosine
IPA: 3-Indolepropionic acid
iTO: Immune-enhanced tumor organoid
MDSC: Myeloid derived suppressor cells
ORR: Overall response rate
PYO: Pyocyanin
PTO: Patient-derived tumor organoid
SB: Sodium butyrate
SCFA: Short-chain fatty acid
TME: Tumor microenvironment
