# Supplementary material for: Immuno-reactive cancer organoid model to assess effects of the microbiome on cancer immunotherapy
Source: Sci Rep. 2022 Jun 15;12:9983. doi: 10.1038/s41598-022-13930-7 (PMC9200712; doi:10.1038/s41598-022-13930-7)
Supplement: Supplementary file 1 — Supplementary Figures. [file 41598_2022_13930_MOESM1_ESM.docx]

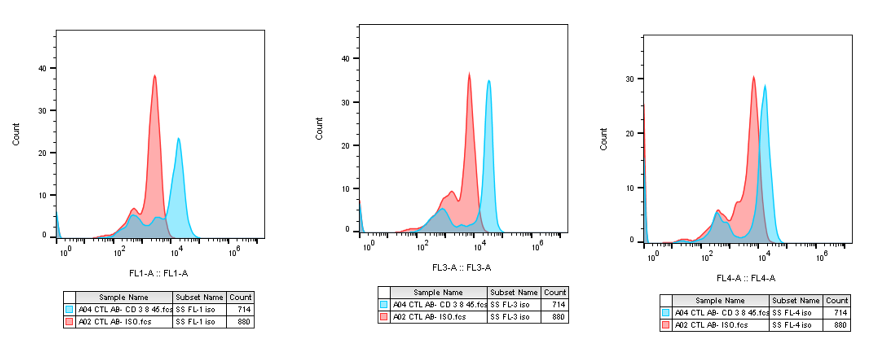


**Supplemental Figure S1.**

**Isotype control graphs for flow cytometry gating.** An isotype control antibody derived from the same host species, utilizing the same Ig class, and linked to the same fluorophore were run in control samples. Gates were placed in flow cytometry to exclude non-specific binding and reduce artifacts. Both forward scatter and side scatter doublet elimination was performed to reduce further noise that could have arisen from organoid digestion.


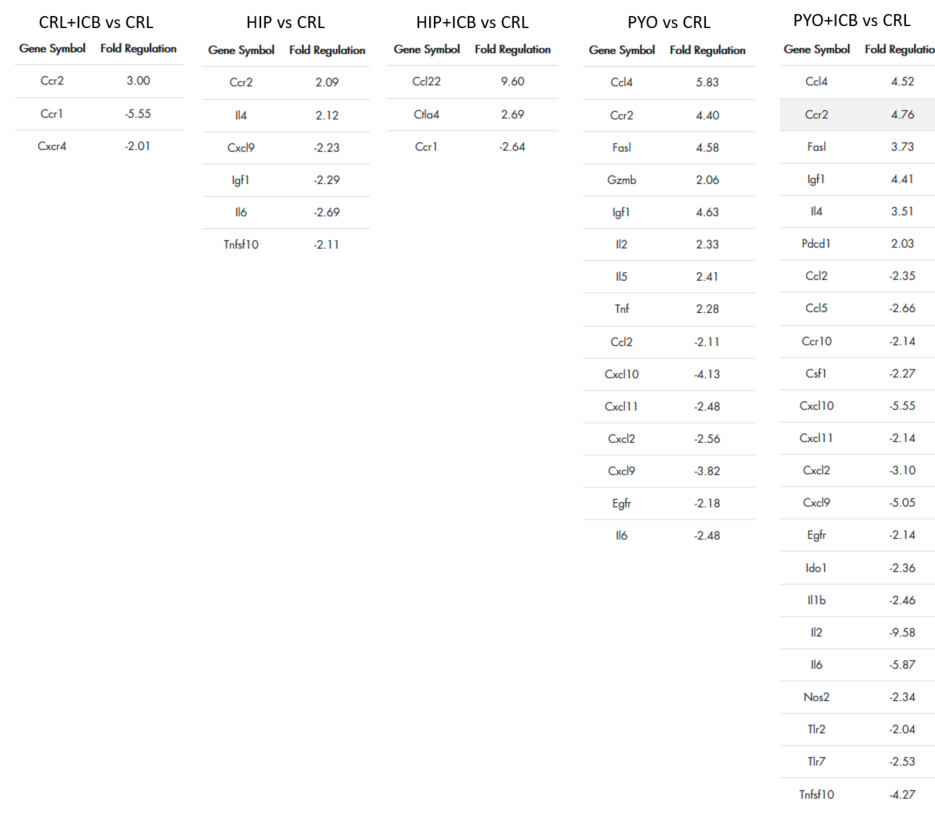


**Supplemental Figure S2.**

**Fold regulation of mRNA expression in HIP and PYO samples with and without ICB compared to untreated CRL group.** Administration of different metabolites had a significant effect on the expression of a wide array of genes. The fold changes for each condition compared to an untreated control are listed above. PYO seemed to have the greatest effect on gene expression. Only PYO and HIP were examined due to assay constraints.

Immuno-Reactive Cancer Organoid Model to Assess Effects of the Microbiome on Cancer Immunotherapy

Ethan Shelkey^1^, David Oommen^2^, Elizabeth R. Stirling^1^, David R. Soto-Pantoja^1^, Katherine L. Cook^1^, Yong Lu^3^, Konstantinos I. Votanopoulos^1^, Shay Soker^1*^

^1^ Wake Forest Baptist Medical Center; Winston-Salem, NC 27101

^2^ Rutgers New Jersey Medical School; Newark, NJ 07103

^3^ Houston Methodist Research Institute; Houston, TX 77030

*Primary correspondence to:

Shay Soker

391 Technology Way

Winston-Salem, NC 27101

ssoker@wakehealth.edu
